# Osteogenic and Anti-Inflammatory Effects of Strontium-Loaded Polydopamine on Micro-Arc Oxidized Titanium Surfaces

**DOI:** 10.3390/jfb17040181

**Published:** 2026-04-07

**Authors:** Yiming Yang, Rongpu Liu, Yuqi Zhou, Lingjun Yuan, Zhenxia Li, Qian Liao, Bing Fang

**Affiliations:** 1 College of Stomatology, Shanghai Jiao Tong University, Shanghai 200011, China; yymsherrya@hotmail.com (Y.Y.); rainbollau@gmail.com (R.L.); yuanlingjun00@126.com (L.Y.); lizhenxiairis@163.com (Z.L.); 2 National Center for Stomatology, National Clinical Research Center for Oral Diseases, Shanghai 200011, China; 3 Shanghai Key Laboratory of Stomatology, Shanghai 200011, China; 4 Shanghai Research Institute of Stomatology, Shanghai 200011, China; 5 Department of Orthodontics, Shanghai Ninth People’s Hospital, School of Medicine, Shanghai Jiao Tong University, Shanghai 200011, China; 6 Department of Prosthodontics, Shanghai Ninth People’s Hospital, School of Medicine, Shanghai Jiao Tong University, Shanghai 200011, China; 7 Department of Implant Alveolar Surgery, The Stomatological Center of Weifang People’s Hospital, Weifang 261000, China; yyu-q@foxmail.com; 8 Department of General Dentistry, Shanghai Ninth People’s Hospital, School of Medicine, Shanghai Jiao Tong University, Shanghai 200011, China

**Keywords:** Micro-Arc Oxidation, Polydopamine, Strontium, Osteogenesis, Anti-inflammatory

## Abstract

Titanium implants are widely used in orthopedic and dental fields but often face challenges such as insufficient osseointegration and peri-implant inflammation. While Strontium (Sr) possesses potent bioactive properties, achieving its controlled delivery at the implant-tissue interface remains technically challenging. To address this, we engineered a multidimensional composite coating by constructing a micro/nano-porous TiO_2_ substrate via micro-arc oxidation (MAO), followed by polydopamine (PDA)-assisted Sr immobilization. This integrated architecture significantly enhanced surface hydrophilicity and facilitated high-content Sr loading with sustained release kinetics. Biological evaluations demonstrated that the PDA-mediated interface promoted superior initial adhesion and spreading of bone marrow mesenchymal stem cells (BMSCs), synergizing with released Sr^2+^ to markedly upregulate core osteogenic markers (*Runx2*, *ALP*). Crucially, the functionalized surface actively optimized the immune microenvironment by inducing M1-to-M2 macrophage polarization and comprehensively suppressing RANKL-induced osteoclastogenesis via the downregulation of TRAP and DC-STAMP. By integrating these pro-osteogenic, anti-inflammatory, and anti-resorptive capabilities, this tri-functional system effectively rebalances the bone remodeling microenvironment. Consequently, it provides a robust, universally applicable strategy for enhancing the therapeutic efficacy of next-generation orthopedic and dental implants.

## 1. Introduction

Titanium and its alloys have become the gold standard for dentistry and orthopedics due to their excellent mechanical properties and biocompatibility [1]. However, the long-term clinical success of these implants is often compromised by insufficient osseointegration and uncontrolled peri-implant inflammation [2,3]. The integration of an implant into host bone is a complex biological process that involves not only the formation of new bone by osteoblasts but also the regulation of host immune responses and bone resorption activities. Therefore, developing a surface modification strategy that can simultaneously enhance osteogenesis, mitigate inflammation, and inhibit osteoclastogenesis is critical for improving therapeutic outcomes.

Strontium (Sr), a trace element naturally present in the human skeleton, has garnered significant attention as a dual-action therapeutic agent. Unlike traditional osteoinductive factors, Sr^2+^ possesses the unique ability to uncouple bone remodeling [4,5,6]—simultaneously stimulating osteoblastic bone formation while suppressing osteoclastic bone resorption [7,8,9]. Despite its potent bioactivity, incorporating Sr^2+^ onto titanium surfaces remains technically challenging. Current strontium delivery strategies predominantly rely on either direct surface deposition or bulk incorporation [10,11,12,13]; however, the former often fails to achieve stable immobilization [12], while the latter results in excessively slow ion diffusion [13]. Ultimately, both approaches fail to maintain the sustained functional concentrations of Sr^2+^ required for long-term therapeutic healing. Consequently, there is an urgent need for an advanced carrier system capable of firmly immobilizing Sr^2+^ and achieving controlled, sustained release at the implant-tissue interface.

To address these challenges, Micro-Arc Oxidation (MAO) and Polydopamine (PDA) chemistry offer a promising combined solution. MAO can fabricate porous, micro/nano-structured titanium oxide coatings that mimic the natural topography of bone, providing a high specific surface area for drug loading [14,15]. Furthermore, PDA, inspired by mussel adhesive proteins, serves as a versatile “bio-glue” and a functional platform for secondary functionalization [16,17,18]. Its abundant catechol and amine groups enable powerful metal ion chelation and versatile interlayer bonding, serving as a stable reservoir to regulate the release kinetics of bioactive agents like Sr^2+^ [19,20,21].

**Figure 1:**
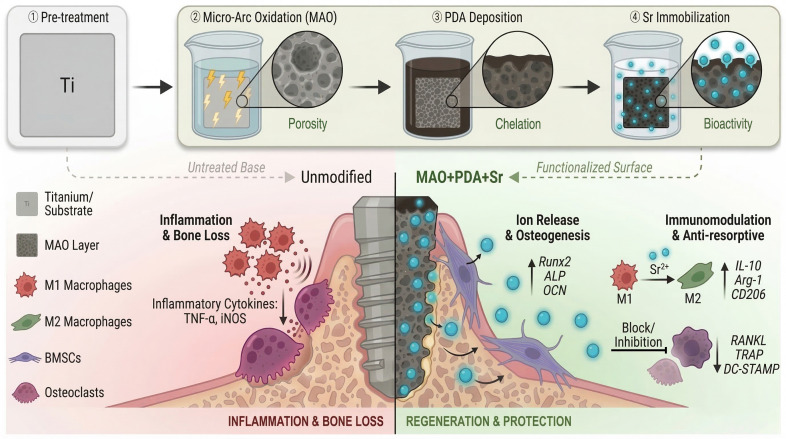
Schematic illustration of the tri-functional MAO+PDA+Sr composite coating designed to orchestrate the osteoimmune microenvironment and rebalance bone remodeling.

In this study, we engineered a tri-functional system by integrating a micro/nano-porous TiO_2_ substrate via MAO as a spatial reservoir, coupled with a PDA chelating layer as a stable interface, thereby achieving highly efficient immobilization and sustained release of Sr^2+^. We hypothesize that this hierarchical architecture creates a favorable microenvironment that rebalances bone remodeling. specifically, we aim to demonstrate that this surface can: (1) enhance the initial adhesion and osteogenic differentiation of bone marrow mesenchymal stem cells (BMSCs); (2) actively optimize the immune microenvironment by promoting M1-to-M2 macrophage polarization; and (3) suppress RANKL-induced osteoclastogenesis. This study provides a comprehensive strategy for overcoming the limitations of current titanium implants by integrating pro-osteogenic, anti-inflammatory, and anti-resorptive capabilities (Figure 1).

## 2. Materials and Methods

### 2.1. Preparation of Materials

The titanium sheets utilized in this study, with dimensions of 10 mm × 10 mm × 1 mm, were made of commercially pure titanium. Initially, the surfaces of the titanium sheets were sanded and polished using fine-grade sandpaper. Subsequently, the sheets were sequentially immersed in acetone, anhydrous ethanol, and deionized water, followed by ultrasonic cleaning for 20 min in each solution. After cleaning, the titanium sheets were dried in an oven at 60 °C for 30 min and stored for subsequent procedures. To achieve nanoporous surface structures, the samples were immersed in an electrolyte solution containing 35.2 g/L calcium acetate monohydrate (C_4_H_6_CaO_4_·H_2_O, Macklin, Shanghai, China) and 12.24 g/L sodium β-glycerophosphate pentahydrate (C_3_H_7_Na_2_O_6_P·5H_2_O, Aladdin, Shanghai, China). MAO was employed to prepare the samples at 360 V, 200 Hz, 30% duty cycle, for 2 min. Subsequently, 18 mM strontium acetate (C_4_H_6_O_4_Sr, Aladdin, Shanghai, China) were added to the aforementioned electrolyte, and the same MAO process was applied to produce the MAO/Sr samples [22,23]. Dopamine hydrochloride powder (DA, Aladdin, Shanghai, China) was dissolved in 10 mM Tris-HCl buffer solution (pH 8.5, Aladdin, Shanghai, China) and thoroughly stirred to form a 2 mg/mL dopamine solution. The MAO samples were submerged in the dopamine solution for 24 h in the dark to fabricate MAO titanium sheets with a PDA coating. Similarly, both the MAO and MAO+PDA samples were immersed in a strontium chloride solution at 18 mM (SrCl_2_, Aladdin, Shanghai, China) for 24 h to generate the MAO+Sr and MAO+PDA+Sr sample groups, respectively.

### 2.2. Material Surface Characteristics

To investigate the physical properties of the material surface, the surface morphology of the micro-arc oxidation coating was examined using a scanning electron microscope (SEM; ZEISS Gemini 300, Oberkochen, Germany). Fiji (ImageJ) software (version 2.14.0, National Institutes of Health, Bethesda, MD, USA)was employed to analyze the pore size and distribution of the coating. The elemental distribution on the material surface was characterized using X-ray energy-dispersive spectroscopy (EDS; Oxford Xplore 300, Abingdon, UK). Surface roughness was evaluated using atomic force microscopy (AFM; Bruker Dimension Icon, Karlsruhe, Germany). The wetting behavior of the coatings was characterized by static contact angle measurements using a contact angle meter (Chengde Dingsheng JY-82C, Chengde, China).

### 2.3. Strontium Ion Release

Titanium slices from the MAO/Sr, MAO+Sr, and MAO+PDA+Sr groups were immersed in the same volume of simulated body fluid (SBF; Phygene, Fuzhou, China) and maintained at room temperature. The solutions were collected on days 1, 3, and 7, and an equal volume of fresh SBF was replenished after each collection to maintain the immersion volume. The concentration of released Sr^2+^ was quantified using an inductively coupled plasma optical emission spectrometer (ICP-OES; Agilent 7850, Santa Clara, CA, USA).

### 2.4. Cell Culture

The rat bone marrow mesenchymal stem cells (rBMSCs) were purchased from ShareBio (Shanghai, China). Cells were cultured in MEM α medium (BasalMedia, L570KJ, Shanghai, China), based on Eagle’s basal medium (BME), supplemented with 10% fetal bovine serum (Every Green, 11011-8611, Hangzhou, China) and 1% penicillin/streptomycin (Biosharp, BL505A, Hefei, China). The culture medium was refreshed every three days, and cells were passaged upon reaching 80–90% confluence. Passage 2–4 rBMSCs were used for subsequent experiments. The RAW264.7 cell line was obtained from the Chinese Academy of Sciences (Shanghai, China). These cells were also cultured in MEM α medium containing 10% fetal bovine serum and 1% penicillin/streptomycin, with the medium replaced every other day. Cells were passaged upon reaching 80% confluence. Both rBMSCs and RAW264.7 cells were maintained in an incubator at 37 °C, 95% humidity and 5% CO_2_.

### 2.5. Cell Counting Kit-8 (CCK-8) Assay and Live/Dead Fluorescence Staining

The biocompatibility of each sample was assessed using the CCK-8 Cell Activity Assay Kit (DOJINDO, Kumamoto, Japan). P3 rBMSCs were inoculated at a density of 5 × 10^4^ cells per well in 24-well plates containing five experimental groups: MAO, MAO/Sr, MAO+Sr, MAO+PDA, and MAO+PDA+Sr. The culture medium was refreshed every two days, and by the third day, the cells had reached confluence in each well. Subsequently, the medium was discarded, and the wells were washed with phosphate-buffered saline (PBS). A culture medium supplemented with 10% CCK-8 solution was then added to each well. Following a 2-h incubation at 37 °C in the dark, the colored culture medium was transferred to 96-well plates, with four replicates per group. The absorbance was measured at 450 nm using a microplate reader, and the resulting data were analyzed to evaluate cytotoxicity. Additionally, a separate batch of rBMSCs was inoculated into 24-well plates with the same five experimental groups at a density of 3 × 10^4^ cells per well, with the culture medium also changed every two days. Following 1, 3, and 7 days of incubation, the culture solution was mixed with CCK-8 reagent (DOJINDO, Kumamoto, Japan) at a 10:1 ratio, subsequently added to the 24-well plates, and incubated for an additional 2 h. The resulting colored culture solution was then transferred to 96-well plates at a volume of 100 μL per well. Absorbance was measured at 450 nm using an enzyme analyzer (Tecan, Männedorf, Switzerland), and the obtained data were used to assess the impact of each sample group on cell proliferation. Additionally, the proliferation of rBMSCs on the surface of galvanized micro-arc titanium oxide was evaluated through live/dead cell staining. rBMSCs were seeded at a density of 3 × 10^4^ cells per well in 24-well plates, each containing one of the five sample groups. The culture medium was refreshed every other day. On days 1, 3, and 5, the supernatant was removed, the wells were rinsed with PBS, and the Calcein/PI cell viability and cytotoxicity assay reagent (Beyotime, C2015S-3, Shanghai, China) was added. The cells were incubated at 37 °C, shielded from light, for 30 min, subsequently washed with PBS, and examined using a fluorescence microscope (Zeiss, Oberkochen, Switzerland) to evaluate the fluorescence staining of live and dead rBMSCs. Image analysis and quantification were conducted using ImageJ software.

### 2.6. Cell Adhesion Assay

For the cell adhesion assay, rBMSCs were seeded onto the surface of each sample group at a density of 3 × 10^4^ cells per well. The culture medium was refreshed every other day, and on the third day, it was aspirated. One group of samples was fixed with pre-cooled 2.5% glutaraldehyde at 4 °C for 2 h. The surfaces of the samples were subsequently rinsed with PBS. Dehydration was performed using a series of graded alcohols (30%, 50%, 70%, 80%, 90%, 95%, and 100%), with each concentration applied twice for 15 min. Following dehydration, the samples were placed in a desiccator (Leica, EM CPD300, Wetzlar, Germany) for critical point drying. After drying, the samples were sputter-coated with gold to enhance conductivity. Upon completion of the preparation, the samples were imaged using a scanning electron microscope (Hitachi S-3400N, Tokyo, Japan). For a separate set of samples, cells were cultured on the substrates for three days, subsequently fixed with 4% paraformaldehyde for 30 min, and rinsed with PBS. This was followed by permeabilization with 0.5% Triton X-100 for 20 min and three additional PBS washes. The cells were then blocked with 5% bovine serum albumin (BSA) for 1 h and incubated overnight at 4 °C with the primary antibody against the adhesion protein vinculin (Abcam, Cat. No. ab11194, Cambridge, UK). The following day, the primary antibody was removed, and the samples were washed three times with PBS. Subsequently, the samples were incubated at 37 °C, protected from light, with Alexa Fluor 488 AffiniPure Donkey Anti-Rabbit IgG (H+L) secondary antibody (Yeasen, Cat. No. 33107ES60, Shanghai, China) for 1 h, followed by a 30-min incubation with TRITC-labeled phalloidin to stain the cytoskeleton. The nuclei were counterstained with DAPI dihydrochloride (Yeasen, Cat. No. 40728ES03, Shanghai, China) for 5 min. Images were captured using a fluorescence inverted microscope (Zeiss, Oberkochen, Germany) for analysis.

### 2.7. Cell Migration Assay

The experimental setup involved placing five distinct material groups at the base of the lower chamber within a Transwell culture system. To this chamber, 800 μL of α-MEM culture medium supplemented with 10% fetal bovine serum (FBS) was added. Subsequently, Transwell inserts were positioned, and 200 μL of a cell suspension containing 5 × 10^4^ rBMSCs was introduced into the upper chamber. Following a 48-h incubation period, the inserts were removed and rinsed with PBS. The lower surfaces of the inserts were then immersed in 4% paraformaldehyde solution for fixation, lasting 30 min. Post-fixation, the inserts were again washed with PBS, followed by staining with 10% crystal violet dye solution (Beyotime, Shanghai, China) for 10 min. The dye was subsequently removed, and the polycarbonate (PC) membranes of the inserts were washed until no residual purple coloration was visible. Cells adhering to the upper side of the inserts were gently removed using a cotton swab. The inserts were then inverted and examined under a light microscope (Zeiss, Oberkochen, Germany) to capture images of cells that had migrated through the PC membrane to the lower side. This assay was conducted to investigate the influence of the components present in the lower chamber’s culture medium on rBMSC migration.

### 2.8. Alkaline Phosphatase (ALP) Activity Analysis

The BCIP/NBT alkaline phosphatase color development assay and the alkaline phosphatase assay were employed to evaluate the impact of sample materials on the osteogenic activity of bone marrow mesenchymal stem cells. rBMSCs were seeded onto titanium sample sheets at a density of 3 × 10^4^ cells per well. The culture medium was refreshed every other day, and samples were harvested after seven days of incubation. The osteogenic activity of rBMSCs was assessed using an alkaline phosphatase color development kit and an alkaline phosphatase assay kit, both purchased from Beyotime, Shanghai, China. The results of the color development assay were examined under a stereomicroscope (Olympus, Tokyo, Japan) to evaluate staining, while the alkaline phosphatase assay was quantified using an enzyme-labeled instrument (Tecan, Männedorf, Switzerland). The optical density (OD) values for *ALP* activity were recorded at a wavelength of 405 nm, and the protein concentration of the samples was measured at 562 nm. These measurements were used for comparison to determine the *ALP* activity.

### 2.9. Immunofluorescence Staining

Immunofluorescence staining was conducted to assess the expression of proteins associated with osteogenesis and inflammation. For the evaluation of osteogenesis-related proteins, namely Runt-related transcription factor 2 (*Runx2*), osteocalcin (*OCN*), and *ALP*, rBMSCs were seeded onto titanium substrates at a density of 3 × 10^4^ cells per well. These cells were cultured with the medium renewed every other day and harvested after a 5-day incubation period. In the assessment of inflammation-associated proteins, specifically those indicative of the M1 phenotype (inducible nitric oxide synthase [iNOS]) and the M2 phenotype (mannose receptor C-type 1 [MRC1; CD206]), RAW264.7 macrophages were seeded onto titanium substrates at a density of 5 × 10^4^ cells per well. Following cell adhesion, the culture medium was replaced with a medium containing 200 ng/mL lipopolysaccharide (LPS; PeproTech, Rocky Hill, NJ, USA) to simulate an inflammatory microenvironment. The medium was refreshed every other day, and the samples were harvested after a 3-day incubation period.

Both cell types underwent identical post-harvest treatments, which included fixation in 4% paraformaldehyde for 30 min, followed by three rinses with PBS. The cells were then permeabilized using 0.5% Triton X-100 for 20 min and washed three additional times with PBS. Subsequently, the cells were blocked with 5% BSA for one hour and then incubated overnight at 4 °C with specific primary antibodies, including anti-Runx2 (Cell Signaling Technology, Cat. No. 12556, Danvers, MA, USA), anti-OCN (Proteintech, Cat. No. 23418, Rosemont, IL, USA), anti-*ALP* (Santa Cruz Biotechnology, Inc., Cat. No. sc-365765, Dallas, TX, USA), anti-iNOS (Santa Cruz Biotechnology, Inc., Cat. No. sc-7271, Dallas, TX, USA), and anti-CD206 (Abcam, Cat. No. ab182112, Cambridge, UK). On the following day, Alexa Fluor 488 AffiniPure Goat Anti-Rabbit IgG (H+L) (Yesean, Cat. No. 33106ES60, Shanghai, China) was utilized for the *CD206*, *Runx2*, and *OCN* groups, while Alexa Fluor 488-AffiniPure Goat Anti-Mouse IgG (H+L) (Yesean, Cat. No. 33206ES60, Shanghai, China) was applied to the iNOS and *ALP* groups, followed by incubation at 37 °C in the dark for 1 h. Finally, the cells were stained with TRITC-labeled phalloidin (Yasen, Cat. No. 40734ES75, Shanghai, China) and DAPI (Yeaten, Cat. No. 40728ES03, Shanghai, China) for 30 min and 5 min, respectively. Fluorescence images were acquired using a fluorescence inverted microscope (Zeiss, Germany) to facilitate both qualitative and quantitative analyses of protein expression levels.

### 2.10. Related Osteogenic Gene Expression

The expression levels of osteogenic genes were assessed using real-time fluorescence polymerase chain reaction (RT-qPCR). rBMSCs were seeded into 24-well plates containing the experimental materials at a density of 3 × 10^4^ cells per well. The culture medium was replaced every other day. On the seventh day, samples were collected, and total ribonucleic acid (RNA) was extracted using TRIzol reagent. Subsequently, the extracted messenger RNA (mRNA) was reverse-transcribed into complementary deoxyribonucleic acid (cDNA) using a reverse transcription kit. Primer sequences, as detailed in Table S1, were employed for the RT-qPCR analysis. The relative expression levels of osteogenesis-related genes, including *Runx2*, Osterix (*Osx*), *ALP*, and *OCN*, were determined using the 2^−ΔΔCt^ method.

### 2.11. Related Inflammatory Gene Expression

RAW264.7 cells were seeded at a density of 5 × 10^4^ cells per well in 24-well plates containing the specified material. After a 2-h incubation period, allowing for full cell adhesion to the plate surface, the culture medium was replaced with a medium supplemented with 200 ng/mL LPS to mimic an inflammatory milieu. The medium was refreshed every other day, and sample collection was conducted on the third day. Total RNA was extracted from the cells using TRIzol reagent (RNAiso Plus, TaKaRa, Tokyo, Japan). The extracted mRNA was then reverse-transcribed into cDNA using a reverse transcription kit (Hifair^®^ V One-Step RT-gDNA Digestion SuperMix for qPCR, Yesean, Cat. No. 11142ES10, Shanghai, China). RT-qPCR was subsequently performed using the primer sequences detailed in Table S2. Glyceraldehyde-3-phosphate dehydrogenase (GAPDH) was used as the reference gene for normalization. The relative expression levels of inducible nitric oxide synthase (*iNOS*), cluster of differentiation 86 (*CD86*), C-C chemokine receptor type 7 (*CCR7*), tumor necrosis factor-alpha (*TNF-α*), cluster of differentiation 163 (*CD163*), mannose receptor C-type 1 (*CD206*), arginase-1 (*Arg-1*), and bone morphogenetic protein 2 (*BMP-2*) were determined using the 2^−ΔΔCt^ method.

### 2.12. Related Osteoclastogenic Gene Expression and Tartrate-Resistant Acid Phosphatase (TRAP) Staining

A total of 3 × 10^5^ RAW264.7 cells per well were seeded into 6-well plates containing three distinct material groups: MAO, MAO+PDA, and MAO+PDA+Sr. Once the cells adhered to the substrate, α-MEM culture medium supplemented with 200 ng/mL LPS was added. After a 2-day incubation period, the supernatant was collected, centrifuged, and filtered to obtain the conditioned culture medium for osteoclast induction. Subsequently, 1 × 10^5^ RAW264.7 cells per well were seeded into 6-well plates, and following cell adhesion, the conditioned medium derived from the aforementioned material groups was used to replace the original medium. Concurrently, receptor activator of nuclear factor kappa-β ligand (RANKL) cytokines at a concentration of 50 ng/mL was added to induce the differentiation of RAW264.7 cells into osteoclasts. The medium was refreshed every other day, and samples were harvested on the fifth day of induction. Total cellular RNA was extracted using the TRIzol method, reverse-transcribed into cDNA, and analyzed via RT-qPCR to assess the expression of osteoclastogenesis-related genes. The primer sequences are provided in Table S3.

Furthermore, RAW264.7 cells were seeded at a density of 1 × 10^4^ cells per well into 96-well plates. Following cell adhesion, osteoclast differentiation was initiated by replacing the medium with three different sets of conditioned media, each containing 50 ng/mL RANKL. The culture medium was refreshed every other day. On the fifth day, the cells were meticulously examined under a microscope. Upon confirmation of increased osteoclast presence, samples were collected and subjected to TRAP staining, following the protocol outlined in the TRAP Staining Kit (Sigma-Aldrich, St. Louis, MO, USA).

### 2.13. Western Blotting for Inflammation-Associated Protein Expression

The expression of inflammation-associated proteins was analyzed using Western blotting. RAW264.7 cells were seeded at a density of 3 × 10^5^ cells per well in 6-well plates containing three distinct material groups: MAO, MAO+PDA, and MAO+PDA+Sr. Once the cells adhered to the plates, LPS was added to stimulate the cells. On the third day of cell culture, total protein was extracted consistently. Subsequently, protein electrophoresis, membrane transfer, and blocking procedures were performed sequentially. Primary antibodies targeting Arg-1, CD206, CD86, and Tubulin were incubated with the membranes overnight at 4 °C. On the following day, secondary antibodies were added, and enhanced chemiluminescence (ECL) was utilized for development and imaging.

### 2.14. Statistical Analysis

Quantitative data are presented as the mean ± standard deviation (SD), derived from at least three independent experiments. Statistical analyses were performed using GraphPad Prism 9.0 software (GraphPad Software, San Diego, CA, USA). Intergroup differences were evaluated by one-way or two-way analysis of variance (ANOVA), followed by Tukey’s post hoc test for multiple comparisons. Statistical significance was set at a threshold of *p* < 0.05, with notations as follows: * for *p* < 0.05, ** for *p* < 0.01, *** for *p* < 0.001, and **** for *p* < 0.0001. The term “ns” indicates non-significant results

## 3. Results

### 3.1. Surface Characterization and In Vitro Sr^2+^ Release Behavior of the Functionalized Titanium Coatings

**Figure 2:**
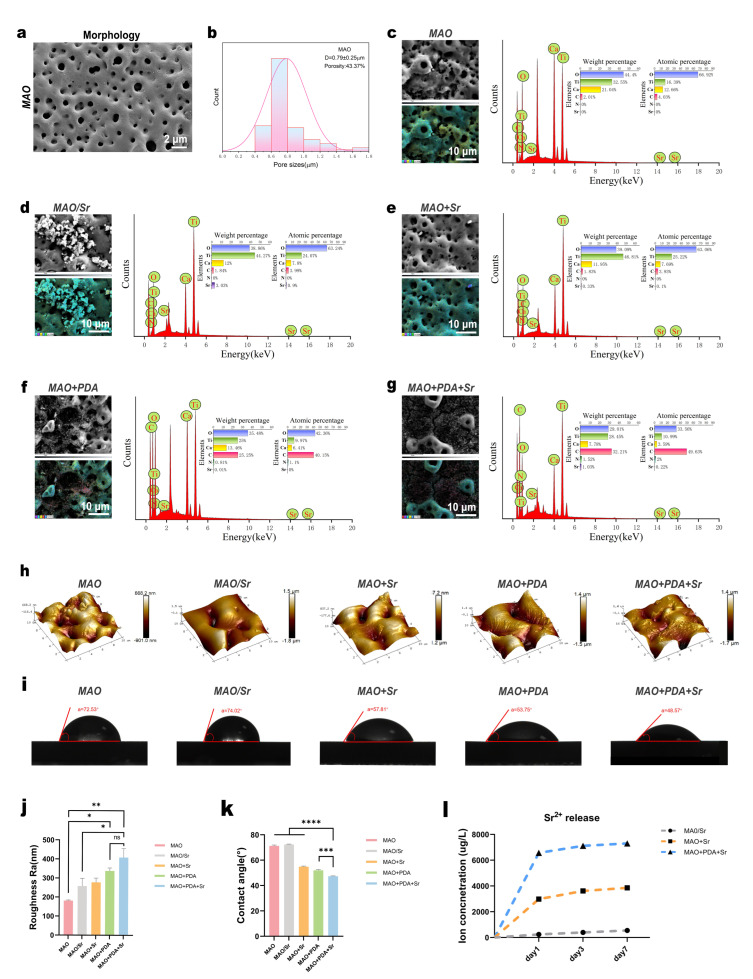
Surface characterization and in vitro Sr^2+^ release behavior of the modified coatings. (**a**) Representative SEM image showing the surface morphology of the MAO coating. (**b**) Quantitative analysis of the surface pore size distribution. (**c**–**g**) EDS elemental analysis results for the five groups. (**h,j**) Three-dimensional (3D) surface topographies (**h**) and the corresponding quantitative surface roughness (**j**) of the five groups. (**i**,**k**) Representative water contact angle images (**i**) and their quantitative measurements (**k**). (**l**) Cumulative Sr^2+^ release profiles of the MAO/Sr, MAO+Sr, and MAO+PDA+Sr groups over 1, 3, and 7 days, as determined by ICP-OES. Data are presented as mean ± SD (*n* = 3). Statistical significance was determined using one-way ANOVA with Tukey’s post hoc test. * *p* < 0.05, ** *p* < 0.01, *** *p* < 0.001, **** *p* < 0.0001; ns, not significant.

Titanium substrates were surface-modified via MAO technology, with subsequent functionalization using PDA and Sr^2+^. SEM observations revealed the formation of hierarchical micro/nano-porous structures across all samples, providing essential transport channels for nutrient exchange and cell infiltration (Figure 2a). Quantitative analysis of surface porosity and pore size showed no significant statistical differences among the groups (Figure 2c–f). The incorporation of PDA, a melanin analogue, imparted a characteristic black appearance to the modified samples. Consistently, SEM imaging identified distinct organic deposits on the MAO+PDA and MAO+PDA+Sr surfaces, with noticeable variations in nanoparticle morphology and distribution compared to the MAO group. EDS analysis confirmed the successful immobilization of PDA, as evidenced by the presence of Nitrogen (1.1 wt.% for MAO+PDA and 2.0 wt.% for MAO+PDA+Sr) and elevated Carbon content (40.15 wt.% and 49.63 wt.%, respectively) in the PDA-containing groups, whereas Nitrogen content was undetectable in the remaining samples. Furthermore, the Sr^2+^ content in the MAO/Sr, MAO+Sr, and MAO+PDA+Sr groups was 3.03 wt.%, 0.33 wt.%, and 1.03 wt.%, respectively. Notably, the Sr^2+^ content in the MAO+PDA+Sr group was approximately three times higher than that in the MAO+Sr group, suggesting that the PDA layer enhanced Sr^2+^ adsorption (Figure 2c–g).

AFM analysis demonstrated that the MAO+PDA+Sr group possessed the highest average surface roughness (Ra = 409 nm), significantly exceeding that of the MAO and MAO/Sr groups (Figure 2h,j). This increase in roughness could be attributed to the combined effects of PDA deposition and subsequent Sr incorporation, which together generated a more pronounced micro/nanostructured surface, as also evidenced by the SEM images in Figure S1. The increased roughness, together with the chemical properties of PDA, significantly improved the surface wettability. Consequently, the MAO+PDA+Sr group exhibited the lowest water contact angle (48.57°) compared to the MAO group (72.53°), indicating favorable hydrophilicity (Figure 2i,k). Furthermore, ICP-OES results demonstrated a stable and sustained Sr^2+^ release profile over 7 days in SBF. On day 1, the MAO+PDA+Sr group exhibited a cumulative release of 6571.40 μg/L, which was significantly higher than both the MAO+Sr and MAO/Sr groups (Figure 2l), correlating well with the EDS surface composition data.

### 3.2. Cytocompatibility and Proliferation Assessment of the Functionalized Coatings

**Figure 3:**
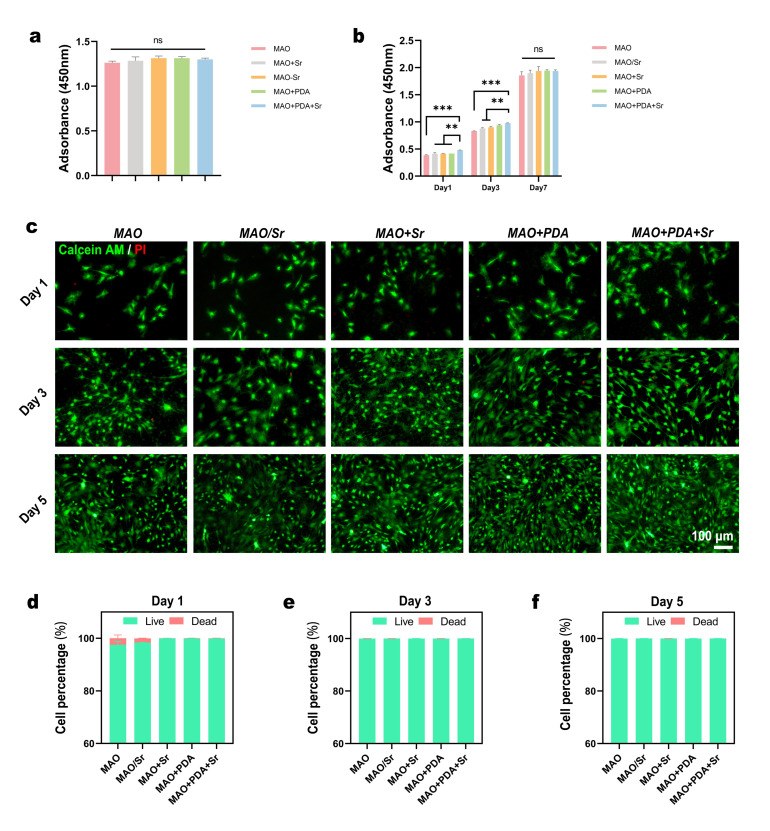
In vitro cytocompatibility of BMSCs cultured on the five groups of samples. (**a**) Cytotoxicity of the various groups evaluated by CCK-8 assay. (**b**) Cell proliferation profiles of BMSCs over 5 days determined by CCK-8 assay. Data are expressed as mean ± SD (*n* = 3). (**c**) Representative fluorescence images of live/dead staining of BMSCs after 1, 3, and 5 days of culture (green: live cells; red: dead cells). (**d**–**f**) Corresponding quantitative analysis of the live-to-dead cell ratios at each time point. For (**a**,**d**–**f**), statistical significance was analyzed using one-way ANOVA with Tukey’s post hoc test. For (**b**), statistical significance was analyzed using two-way ANOVA with Tukey’s post hoc test. ** *p* < 0.01, *** *p* < 0.001; ns, not significant.

To evaluate the potential cytotoxicity of the five experimental groups (MAO, MAO/Sr, MAO+Sr, MAO+PDA, and MAO+PDA+Sr), rBMSCs were initially seeded at a high density onto the material surfaces. The CCK-8 assay performed on day 2 revealed no statistically significant differences in OD values among the groups (Figure 3a). This lack of variation indicates that the modified coatings impose no inhibitory effects on cell growth, demonstrating excellent biocompatibility and non-cytotoxicity.

For the cell proliferation assay, rBMSCs were seeded at a lower density, and their metabolic activity was monitored on days 1, 3, and 7. The results demonstrated that the MAO+PDA+Sr group exhibited significantly higher OD values compared to the MAO, MAO/Sr, and MAO+Sr groups on both day 1 and day 3. Although the MAO+PDA+Sr group showed slightly higher viability than the MAO+PDA group, the difference was not statistically significant (Figure 3b). These findings suggest that during the early phase, the PDA-functionalized surfaces significantly promoted cell attachment and proliferation. By day 7, however, cells in all groups reached confluence, resulting in comparable OD values across all samples, which aligns with the cytotoxicity results observed in the high-density assay.

To further corroborate these findings, Live/Dead fluorescence staining was performed on days 1, 3, and 5. As shown in Figure 3c, rBMSCs on all surfaces displayed a time-dependent increase in density, characterized by a predominance of viable cells (green fluorescence) and negligible dead cells (red fluorescence). Quantitative analysis of the live-to-dead cell ratios (Figure 3d–f) indicated that, with the exception of slightly higher mortality rates in the MAO and MAO/Sr groups on day 1, no significant differences were observed at subsequent time points.

In summary, the composite coatings exhibited excellent biocompatibility and biological safety, effectively supporting the adhesion and proliferation of rBMSCs and creating a favorable microenvironment for subsequent osteogenic differentiation.

### 3.3. Evaluation of Cell Adhesion and Migration Behaviors on the Functionalized Coatings

**Figure 4:**
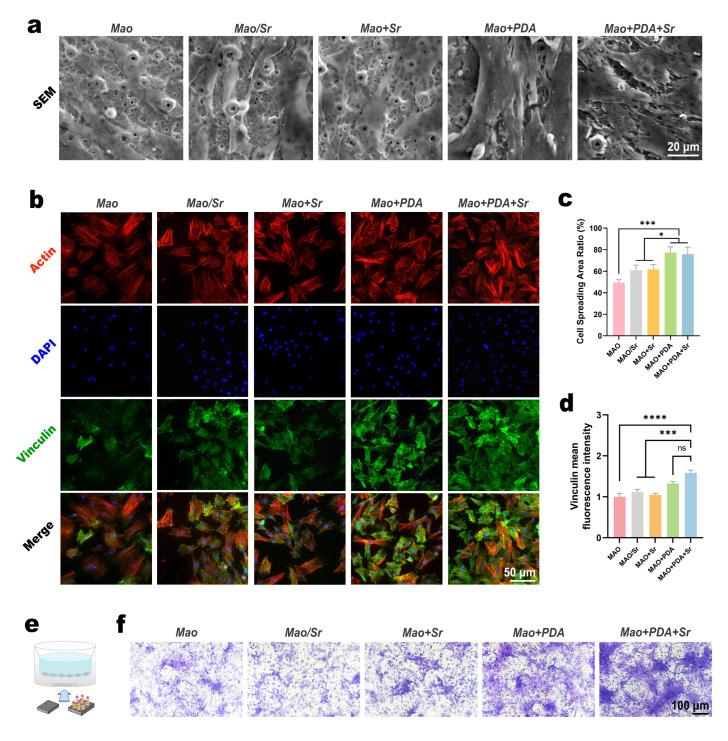
BMSCs adhesion and migration evaluations. (**a**) Representative SEM images of cell spreading morphology. (**b**) Immunofluorescence staining of Vinculin (green), Actin (red) and nuclei (blue). (**c**,**d**) Quantitative analysis of (**c**) cell area and (**d**) Vinculin fluorescence intensity. (**e**) Schematic diagram of the Transwell assay. (**f**) Transwell migration assay results demonstrating the recruitment of BMSCs toward the different groups. Data are mean ± SD (*n* = 3). Analyzed by one-way ANOVA with Tukey’s post hoc test. * *p* < 0.05, *** *p* < 0.001, **** *p* < 0.0001; ns, no significant difference.

To assess the early adhesion behavior of rBMSCs, cells were cultured on the various samples for three days, followed by fixation and SEM observation. As shown in Figure 4a, cells in the MAO, MAO/Sr, and MAO+Sr groups exhibited a spindle-shaped morphology with limited spreading, primarily appearing as isolated individual cells. In contrast, rBMSCs on the PDA-functionalized surfaces (MAO+PDA and MAO+PDA+Sr) displayed a flattened, polygonal morphology with extensive filopodia extension and intercellular connections, indicating superior initial attachment. Quantitative analysis using ImageJ software (Figure 4c) confirmed that the cell spreading areas in the MAO+PDA and MAO+PDA+Sr groups were significantly larger compared to the other three groups.

To further elucidate the molecular mechanism underlying this enhanced adhesion, the expression of vinculin, a key focal adhesion protein, was evaluated by immunofluorescence after three days of culture. Confocal microscopy revealed that the fluorescence intensity of vinculin in the MAO+PDA and MAO+PDA+Sr groups was markedly higher than that in the non-PDA-coated groups (Figure 4b). Quantitative analysis of the mean fluorescence intensity (Figure 4d) demonstrated that the MAO+PDA+Sr group exhibited the highest expression levels, although no statistically significant difference was observed compared to the MAO+PDA group. These results collectively indicate that the introduction of the PDA coating significantly upregulates the formation of focal adhesions, thereby promoting stable cell anchorage.

Furthermore, a Transwell assay was conducted to evaluate the recruitment capability of the modified coatings. The material samples were placed in the lower chamber, while rBMSCs were seeded in the upper chamber. After 48 h, the migrated cells on the lower surface of the polycarbonate membrane were stained with crystal violet. As illustrated in Figure 4e, the MAO+PDA and MAO+PDA+Sr groups significantly enhanced the transmembrane migration of rBMSCs compared to the control groups. Notably, the MAO+PDA+Sr group exhibited the highest number of migrated cells. This enhanced recruitment can be attributed to a combined effect: the PDA layer provides favorable sites for cell adhesion, while the sustained release of Sr^2+^ acts as a chemoattractant, effectively recruiting stem cells to the material surface.

### 3.4. Osteogenic Differentiation Assessment of the Functionalized Coatings

**Figure 5:**
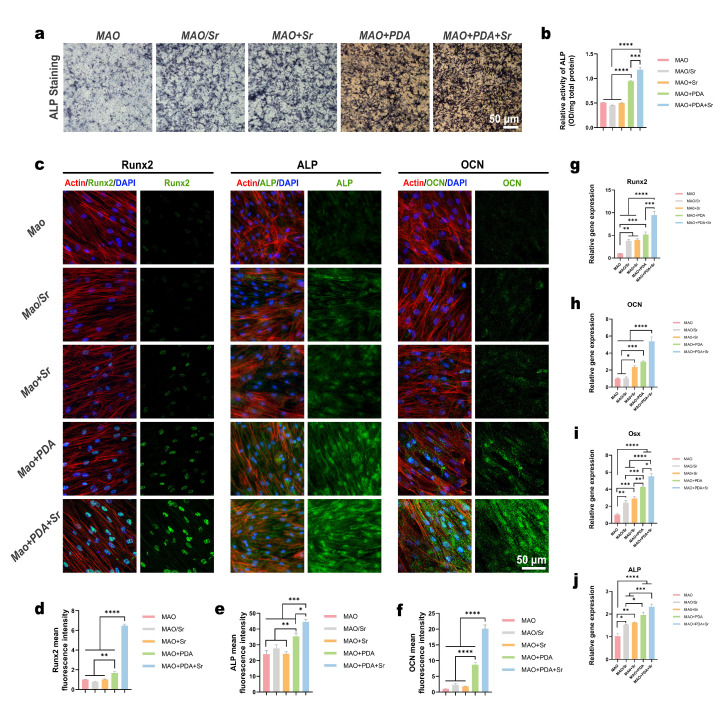
Osteogenic differentiation of BMSCs cultured on different samples. (**a**) Representative images of *ALP* staining of BMSCs in each group after 7 days of induction. (**b**) Quantitative analysis of *ALP* activity based on the colorimetric assay. (**c**) Representative immunofluorescence images showing the expression of *Runx2*, *ALP*, and *OCN*. (**d**–**f**) Quantitative analysis of the relative fluorescence intensities for (**d**) *Runx2*, (**e**) *ALP*, and (**f**) *OCN*. (**g**–**j**) Relative mRNA expression levels of osteogenesis-related genes, including (**g**) *Runx2*, (**h**) *OCN*, (**i**) *Osterix*, and (**j**) *ALP*, determined by RT-qPCR after 7 days of culture. Data are expressed as mean ± SD (*n* = 3). Statistical significance was determined using one-way ANOVA with Tukey’s post hoc test. * *p* < 0.05, ** *p* < 0.01, *** *p* < 0.001, **** *p* < 0.0001.

*ALP* is a well-established early marker of osteogenic differentiation; its elevated activity typically signifies the commitment of pre-osteoblasts toward a mature osteoblastic lineage. To evaluate the osteoinductive potential of the different coatings, rBMSCs were cultured on the titanium surfaces, and *ALP* activity was assessed. Qualitative *ALP* staining revealed that the MAO+PDA+Sr group exhibited the most intense chromogenic reaction, followed by the MAO+PDA group, whereas the MAO+Sr, MAO/Sr, and MAO groups showed relatively weaker staining (Figure 5a). Quantitative analysis further confirmed that *ALP* activity in the PDA-functionalized groups (MAO+PDA and MAO+PDA+Sr) was significantly higher than that in the other three groups (*p* < 0.0001). Notably, the MAO+PDA+Sr group demonstrated a statistically significant increase in *ALP* levels compared to the MAO+PDA group (*p* < 0.001) (Figure 5b).

Furthermore, immunofluorescence staining demonstrated that the MAO+PDA+Sr group exhibited the most intense expression of osteogenic proteins (*Runx2*, *ALP*, and *OCN*) (Figure 5c), which was confirmed by quantitative analysis of fluorescence intensity (Figure 5d–f). To validate these findings at the transcriptional level, the mRNA expression of *Runx2*, *Osx*, *ALP*, and *OCN* was quantified via RT-qPCR. Consistent with the protein data, the MAO+PDA+Sr group induced the highest upregulation of these genes, followed by the MAO+PDA group (Figure 5g–j).

Collectively, these results demonstrate that while both Sr^2+^ doping and PDA coating enhance the osteogenic differentiation of rBMSCs, the dual-functionalized MAO+PDA+Sr surface exerts a combined effect. This optimized microenvironment significantly accelerates the differentiation of stem cells into osteoblasts, making it a highly promising candidate for bone repair applications.

### 3.5. Immunomodulatory Effects and Macrophage Polarization Profiles

**Figure 6:**
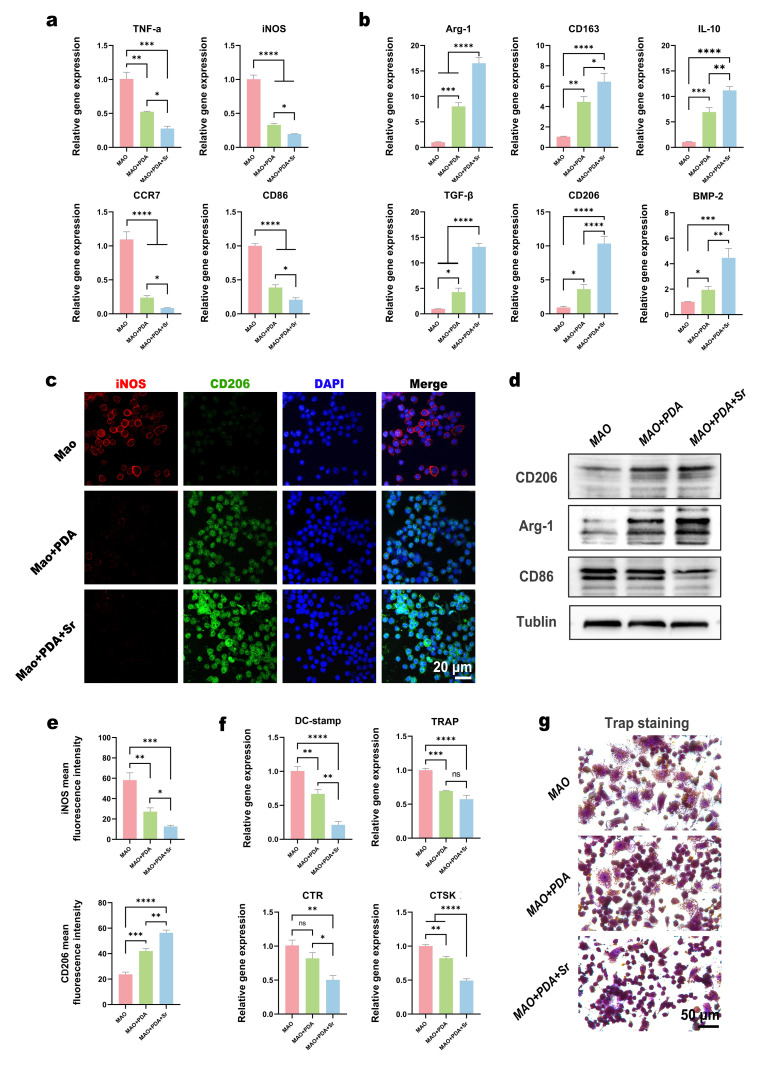
Immunomodulatory effects and osteoclastogenic evaluation of the modified coatings. (**a**,**b**) Relative mRNA expression levels of (**a**) pro-inflammatory and (**b**) inflammation-suppressing (anti-inflammatory) genes in macrophages cultured on the MAO, MAO+PDA, and MAO+PDA+Sr groups, determined by RT-qPCR. (**c**) Representative immunofluorescence double-staining images of the M1 macrophage marker iNOS (red) and M2 macrophage marker CD206 (green) in the indicated groups. (**d**) Representative Western blot images showing the expression of inflammation-related proteins. (**e**) Quantitative analysis of the relative immunofluorescence intensities of CD206 and iNOS. (**f**) RT-qPCR analysis of osteoclastogenesis-related gene expressions. (**g**) Representative TRAP staining images illustrating osteoclast differentiation in the MAO, PDA, and PDA+Sr groups. Data are expressed as mean ± SD (*n* = 3). Statistical significance was determined using one-way ANOVA with Tukey’s post hoc test. * *p* < 0.05, ** *p* < 0.01, *** *p* < 0.001, **** *p* < 0.0001; ns, not significant.

To investigate the immunomodulatory properties of the modified coatings, RAW 264.7 macrophages were cultured on the MAO, MAO+PDA, and MAO+PDA+Sr surfaces and challenged with LPS to simulate a pro-inflammatory microenvironment. After three days of incubation, the transcriptional levels of polarization markers were quantified via RT-qPCR. The results demonstrated a significant downregulation of M1-related pro-inflammatory genes, including *Tnf-α*, *Inos*, *Ccr7*, and *Cd86*, in the MAO+PDA+Sr group compared to the MAO and MAO+PDA groups (Figure 6a). Conversely, the expression of M2-related anti-inflammatory markers—such as *Arg-1*, *Tgf-β*, *Cd163*, *Cd206*, *Il-10*, and *Bmp-2*—was markedly upregulated in the MAO+PDA+Sr group (Figure 6b). These findings suggest that while the PDA coating alone possesses intrinsic anti-inflammatory properties, the sustained release of Sr^2+^ from the MAO+PDA+Sr surface further enhances the transition of macrophages from a pro-inflammatory M1 state to a pro-regenerative M2 phenotype.

To further validate this phenotypic transition at the translational level, immunofluorescence double-staining for iNOS (M1 marker, red) and CD206 (M2 marker, green) was performed. As illustrated in Figure 6c, the MAO group exhibited intense red fluorescence (iNOS), whereas the MAO+PDA+Sr group showed a predominant green fluorescence signal (CD206). Quantitative analysis of the fluorescence intensity confirmed that the MAO+PDA+Sr group significantly suppressed iNOS expression while promoting CD206 expression compared to the other groups (Figure 6e). Consistent with the immunofluorescence findings, Western blot analysis demonstrated that the MAO+PDA+Sr group expressed the highest levels of M2-associated proteins (CD206 and Arg-1) and the lowest levels of the M1-associated protein CD86 (Figure 6d).

In summary, these results collectively indicate that the active components released from the MAO+PDA+Sr coating can effectively remodel the inflammatory microenvironment. By modulating macrophage polarization toward the M2 phenotype, this functionalized surface creates a favorable osteo-immunomodulatory niche that is conducive to subsequent bone tissue regeneration and repair.

### 3.6. Inhibition of Osteoclastogenesis via Immunomodulatory Conditioned Media

To evaluate the influence of the material-modulated immune microenvironment on osteoclast differentiation, conditioned media (CM) were collected from RAW 264.7 cells cultured on the MAO, MAO+PDA, and MAO+PDA+Sr surfaces under LPS stimulation. Subsequently, RAW 264.7 macrophages were cultured in these CM supplemented with RANKL to induce osteoclastogenesis. After five days of induction, the expression of osteoclast-specific genes was quantified by RT-qPCR. The results revealed that the mRNA levels of dendritic cell-specific transmembrane protein (*Dc-stamp*), *TRAP*, calcitonin receptor (*CTR*), and cathepsin K (*CTSK*) were significantly downregulated in the MAO+PDA+Sr group compared to the MAO and MAO+PDA groups (Figure 6f).

Furthermore, TRAP staining was performed to visualize the formation of mature osteoclasts. As illustrated in Figure 6g, numerous large, TRAP-positive multinucleated cells were observed in the MAO group, indicating robust osteoclast differentiation. Notably, the MAO+PDA+Sr group exhibited a pronounced inhibitory effect on osteoclast formation, which was further corroborated by the quantitative analysis of TRAP-positive multinucleated cells presented in Figure S2. These histological observations further corroborated the RT-qPCR findings, confirming that the MAO+PDA+Sr coating effectively inhibits RANKL-induced osteoclastogenesis by modulating the macrophage-mediated inflammatory crosstalk. This suppression of osteoclast activity, combined with the previously observed pro-osteogenic effects, suggests that the MAO+PDA+Sr surface can effectively maintain the balance of bone metabolism.

## 4. Discussion

In this study, we successfully engineered a tri-functional MAO+PDA+Sr composite coating designed to address the clinical bottlenecks of insufficient osseointegration, persistent inflammation, and excessive bone resorption. By integrating the adhesive versatility of PDA with the dual-action bioactivity of Sr^2+^ on a micro/nano-porous MAO substrate, we achieved a coordinated modulation of the osteoimmune microenvironment. Our findings confirm that this hierarchical architecture orchestrates a multidimensional regulatory system—promoting osteogenesis, modulating inflammation, and inhibiting osteoclastogenesis—thereby rebalancing the bone remodeling process. The superior regenerative performance of the MAO+PDA+Sr group is attributed to the physicochemical synergy between the bioactive interface and the efficient delivery of Sr ions to achieve biologically active concentrations (mg/L level) [24]. Leveraging its adhesive properties and reactivity, PDA firmly anchors to the MAO coating surface and facilitates the efficient, sustained release of Sr^2+^ through complexation, ensuring sustained therapeutic kinetics. This controlled delivery establishes a virtuous cycle that actively shifts the microenvironment from a pro-inflammatory state to a regenerative, anti-resorptive state.

Optimizing the surface properties of titanium implants is fundamental to directing cellular behavior and achieving successful osseointegration. Specifically, surface topography and hydrophilicity are critical determinants of initial cell adhesion and subsequent hard tissue regeneration. Previous studies demonstrate that micrometer-scale roughness enhances osteoblast adhesion and differentiation [20], while nanoscale features further augment cellular proliferation, highlighting the efficacy of hierarchical micro/nano-topographies [21]. Consistent with this rationale, the MAO treatment in our study generated a biomimetic hierarchical micro/nano-pitted structure (Figure 2a), providing robust anchorage points for BMSC adhesion (Figure 4a). Notably, as shown in Figure 5a, the titanium surface subjected to MAO treatment alone also exhibited a certain osteogenic-promoting effect, which may be attributed to its rough and porous surface morphology; this observation is consistent with previous literature reports [25,26].

Furthermore, surface hydrophilicity is a pivotal factor mediating protein adsorption and cell-material interactions. While traditional metal ions or native TiO_2_ layers can partially improve surface wettability [27,28], our contact angle measurements revealed that the PDA/Sr^2+^-functionalized interface achieved a hydrophilic state (48.57°, Figure 2i), significantly lower than control groups. This optimized wettability is crucial for facilitating rapid BMSCs spreading and initiating the osteogenic cascade. Furthermore, the abundant catechol and amine groups of PDA form stable coordination complexes with Sr^2+^. This chelation enables controlled, sustained release kinetics, maintaining localized Sr^2+^ concentrations within the optimal range for osteo-induction. This sustained release profile mirrors the controlled delivery achieved by mesoporous bioactive carriers [29]. Thus, our PDA-mediated immobilization successfully resolves the technical challenge of Sr^2+^ delivery, validating “substrate-interface-ion” engineering as a highly effective strategy for titanium implant modification. Beyond its superior biocompatibility, the PDA layer also serves as a protective barrier that enhances the corrosion resistance of the underlying metallic substrate—a critical requirement for ensuring the long-term stability and clinical longevity of orthopedic and dental implants [17,30,31,32].

Biological evaluations confirmed robust osteogenic differentiation in the MAO+PDA+Sr group, evidenced by the marked upregulation of core markers (*Runx2*, *ALP*, *OCN*). Mechanistically, *ALP*, *Runx2*, and *OCN* serve as core markers of osteogenic differentiation: *Runx2*, as a key transcription factor, modulates downstream *ALP* and *OCN* expression to promote bone formation; *ALP* participates in extracellular matrix mineralization, while *OCN* plays a crucial role in bone matrix maturation [33,34].

The enhanced osteogenic performance observed in this study may be related to the combined effects of PDA and Sr^2+^. Previous studies have suggested that PDA coating can improve surface hydrophilicity and biocompatibility, facilitate cell adhesion, promote extracellular matrix protein expression, and potentially support osteogenic differentiation through integrin-mediated cell–matrix interactions [17,35]. Meanwhile, Sr^2+^, as a bioactive osteogenic ion, has been reported to promote osteoblast proliferation and differentiation, possibly through pathways such as FAK/P38, Smad1/5/9, and Wnt/β-catenin [17,35,36,37,38,39]. Therefore, the superior osteogenic capacity of our tri-functional system may be associated with the interplay between surface adhesion-related regulation and Sr^2+^-mediated biochemical signaling, although the underlying mechanisms remain to be further clarified in future studies.

Macrophage polarization is a critical component in regulating the immune microenvironment and tissue repair/regeneration. M1 macrophages mediate proinflammatory responses, while M2 macrophages play a central role in anti-inflammation and promoting tissue repair. This study confirmed via RT-qPCR, immunofluorescence, and Western blot analyses that the MAO+PDA+Sr group significantly suppressed the expression of proinflammatory factors (TNF-α, iNOS, CCR7, CD86), while simultaneously upregulating anti-inflammatory factors (Arg-1, TGF-β, CD163, CD206, IL-10, BMP2). This effectively induced Raw264.7 macrophages to shift from M1 to M2 polarization, establishing an anti-inflammatory microenvironment conducive to osteogenic repair. These findings are in line with previous studies suggesting that PDA and Sr^2+^ may contribute to anti-inflammatory and pro-regenerative effects through NF-κB-related pathways [40,41,42].

Crucially, our system addresses the challenge of excessive bone resorption. The MAO+PDA+Sr group significantly inhibited RANKL-induced osteoclastogenesis, as evidenced by the downregulation of *TRAP*, *CTSK*, and *DC-STAMP*. This aligns with the established “dual-action” of Sr^2+^, which uncouples bone remodeling by upregulating OPG (via LRP6/β-catenin) and suppressing NF-κB-mediated osteoclast activation [43,44,45]. As a classic bone metabolism regulator, Sr^2+^ has been extensively demonstrated to inhibit osteoclast differentiation through two mechanisms: first, by enhancing the LRP6/β-catenin-mediated OPG signaling pathway, indirectly blocking RANKL-induced osteoclast differentiation [43]; second, by suppressing the NF-κB signaling pathway, significantly reducing osteoclast formation and decreasing their resorptive activity [44]. Moreover, Sr^2+^ exerts bidirectional regulation by promoting bone formation while inhibiting resorption, effectively maintaining bone metabolic equilibrium and serving as an effective anti-osteoporosis therapeutic agent [7,46,47].

This study validates a novel “substrate-interface-ion” engineering paradigm for titanium surface modification, confirming that multi-component synergy is essential for optimizing osseointegration. By addressing critical clinical barriers—specifically insufficient osseointegration and excessive osteoclast activation—our findings offer a robust experimental basis for clinical translation. Furthermore, this work elucidates the molecular mechanisms governing the interplay between surface chemistry and the osteoimmune microenvironment. Collectively, these insights enrich our understanding of surface-mediated bone metabolism, providing a theoretical framework for future fundamental and applied research in bone tissue engineering.

Despite these promising results, several limitations warrant consideration. First, the reliance on in vitro models limits our ability to fully recapitulate the systemic complexity of the in vivo physiological environment. Second, the long-term release kinetics of Sr^2+^ and its potential late-stage biological effects require further characterization to ensure sustained therapeutic efficacy. Future investigations will prioritize validating these findings in large animal translational models (e.g., porcine or ovine defects) to comprehensively assess long-term osseointegration. Concurrently, we aim to expand this PDA-based platform to explore the enhanced co-delivery of other therapeutic ions (e.g., Mg, Zn), further elucidating multi-ion regulatory networks in bone repair.

From a translational perspective, the integration of precise multifunctional regulation positions this composite coating as a highly promising candidate for managing complex clinical challenges, such as osteoporosis-related bone defects. Given its distinct versatility, this surface engineering approach can be seamlessly coupled with advanced manufacturing technologies, such as 3D-printed porous scaffolds and personalized implants. Therefore, the “substrate-interface-ion” strategy serves not merely as a localized treatment modality, but as a highly adaptable platform for advancing the broader field of implantology and bone tissue engineering.

## 5. Conclusions

In summary, our results demonstrate that we successfully engineered a trifunctional MAO+PDA+Sr composite coating with favorable biocompatibility, and that it exhibits notable osteoinductive, immunomodulatory and anti-resorptive properties. By addressing the inherent limitations of traditional single-component coatings in sustaining long-term effective therapeutic ion concentrations, the proposed substrate–interface–ion strategy enables the coordinated modulation of the osteoimmunomodulatory microenvironment in our experimental models. Consistent with the current osteoimmunomodulation paradigm, this multifunctional platform may disrupt aberrant bone remodeling and thus holds the potential to promote bone regeneration. Ultimately, this system offers a reliable approach for controlled bioactive ion delivery, representing a promising strategy that could enhance long-term implant stability, particularly in compromised bone conditions such as osteoporosis.

## Data Availability

The original contributions presented in the study are included in the article. Further inquiries can be directed to the corresponding author.

## Supplementary Materials

The following supporting information can be downloaded at: https://www.mdpi.com/article/10.3390/jfb17040181/s1, Table S1: Primers for osteogenesis-related gene expression in rBMSCs; Table S2: Primers for inflammation-related gene expression in RAW264.7 cells; Table S3: Primers for osteoclast-related gene expression in mice. Figure S1. Representative SEM images showing the surface morphologies of different coating materials under various background conditions at magnifications of 3000×, 5000× and 10,000×, respectively. Figure S2. Quantitative analysis of osteoclast number in each group stained by TRAP. Statistical significance was determined using one-way ANOVA with Dunnett’s post hoc test. **** *p* < 0.0001; ns, not significant.

## Abbreviations

The following abbreviations are used in this manuscript:

Sr	Strontium
MAO	Micro-Arc Oxidation
PDA	Polydopamine
BMSCs	Bone Marrow Mesenchymal Stem Cells
SEM	Scanning Electron Microscope
EDS	Energy Dispersive Spectroscopy
AFM	Atomic Force Microscopy
SBF	Simulated Body Fluid
ICP-OES	Inductively Coupled Plasma Optical Emission
rBMSCs	rat Bone Marrow Mesenchymal Stem Cells
CCK-8	Cell Counting Kit-8
PBS	Phosphate-Buffered Saline
BSA	Bovine Serum Albumin
ALP	Alkaline Phosphatase
OD	Optical Density
Runx2	Runt-related Transcription Factor2
OCN	Osteocalcin
Osx	Osterix
iNOS	Inducible Nitric Oxide Synthase
CD206	Mannose Receptor C-type 1
LPS	Lipopolysaccharide
RNA	Ribonucleic Acid
mRNA	Messenger Ribonucleic Acid
cDNA	Complementary Deoxyribonucleic Acid
RT-qPCR	Real-Time Fluorescence Polymerase Chain Reaction
CD86	Cluster of Differentiation 86
CCR7	C-C chemokine receptor type 7
TNF-α	Tumor Necrosis Factor-Alpha
CD163	Cluster of Differentiation 163
Arg-1	Arginase-1
BMP-2	Bone Morphogenetic Protein 2
TRAP	Tartrate-Resistant Acid Phosphatase
RANKL	Receptor Activator of Nuclear Kappa-β
CM	Conditioned Media
Dc-STAMP	Dendritic Cell-Specific Transmembrane Protein
CTR	Calcitonin Receptor
CTSK	Cathepsin K

## References

[1] Niinomi M. (1998). Titanium alloys for biomedical applications. Mater. Sci. Eng. A.

[2] Romanos G.E., Fischer G.A., Delgado-Ruiz R. (2021). Titanium Wear of Dental Implants from Placement, under Loading and Maintenance Protocols. Int. J. Mol. Sci..

[3] Di Tinco R., Bertani G., Pisciotta A., Bertoni L., Bertacchini J., Colombari B., Conserva E., Blasi E., Consolo U., Carnevale G. (2021). Evaluation of Antimicrobial Effect of Air-Polishing Treatments and Their Influence on Human Dental Pulp Stem Cells Seeded on Titanium Disks. Int. J. Mol. Sci..

[4] Gentleman E., Fredholm Y.C., Jell G., Lotfibakhshaiesh N., O’Donnell M.D., Hill R.G., Stevens M.M. (2010). The Effects of Strontium-Substituted Bioactive Glasses on Osteoblasts and Osteoclasts in Vitro. Biomaterials.

[5] Saidak Z., Marie P.J. (2012). Strontium Signaling: Molecular Mechanisms and Therapeutic Implications in Osteoporosis. Pharmacol. Ther..

[6] Leite Á.J., Gonçalves A.I., Rodrigues M.T., Gomes M.E., Mano J.F. (2018). Strontium-Doped Bioactive Glass Nanoparticles in Osteogenic Commitment. ACS Appl. Mater. Interfaces.

[7] Peng S., Liu X.S., Zhou G., Li Z., Luk K.D., Guo X.E., Lu W.W. (2011). Osteoprotegerin deficiency attenuates strontium-mediated inhibition of osteoclastogenesis and bone resorption. J. Bone Miner. Res..

[8] Schumacher M., Wagner A.S., Kokesch-Himmelreich J., Bernhardt A., Rohnke M., Wenisch S., Gelinsky M. (2016). Strontium substitution in apatitic CaP cements effectively attenuates osteoclastic resorption but does not inhibit osteoclastogenesis. Acta Biomater..

[9] Schumacher M., Gelinsky M. (2015). Strontium modified calcium phosphate cements—Approaches towards targeted stimulation of bone turnover. J. Mater. Chem. B.

[10] Costa A.I., Gemini-Piperni S., Alves A.C., Costa N.A., Checca N.R., Leite P.E., Rocha L.A., Pinto A.M.P., Toptan F., Rossi A.L. (2021). TiO_2_ bioactive implant surfaces doped with specific amount of Sr modulate mineralization. Mater. Sci. Eng. C.

[11] Shen X., Fang K., Ru Yie K.H., Zhou Z., Shen Y., Wu S., Zhu Y., Deng Z., Ma P., Ma J. (2022). High proportion strontium-doped micro-arc oxidation coatings enhance early osseointegration of titanium in osteoporosis by anti-oxidative stress pathway. Bioact. Mater..

[12] Göttlicher M., Rohnke M., Moryson Y., Thomas J., Sann J., Lode A., Schumacher M., Schmidt R., Pilz S., Gebert A. (2017). Functionalization of Ti-40Nb implant material with strontium by reactive sputtering. Biomater. Res..

[13] Hayashi K., Zhang C., Taleb Alashkar A.N., Ishikawa K. (2024). Carbonate Apatite Honeycomb Scaffold-Based Drug Delivery System for Repairing Osteoporotic Bone Defects. ACS Appl. Mater. Interfaces.

[14] Yao Z.Q., Ivanisenko Y., Diemant T., Caron A., Chuvilin A., Jiang J.Z., Valiev R.Z., Qi M., Fecht H.-J. (2010). Synthesis and Properties of Hydroxyapatite-Containing Porous Titania Coating on Ultrafine-Grained Titanium by Micro-Arc Oxidation. Acta Biomater..

[15] Jing W., Zhang M., Jin L., Zhao J., Gao Q., Ren M., Fan Q. (2015). Assessment of osteoinduction using a porous hydroxyapatite coating prepared by micro-arc oxidation on a new titanium alloy. Int. J. Surg..

[16] Han L., Wang M., Li P., Gan D., Yan L., Xu J., Wang K., Fang L., Chan C.W., Zhang H. (2018). Mussel-inspired tissue-adhesive hydrogel based on the polydopamine-chondroitin sulfate complex for growth-factor-free cartilage regeneration. ACS Appl. Mater. Interfaces.

[17] Wang H., Lin C., Zhang X., Lin K., Wang X., Shen S.G. (2019). Mussel-inspired polydopamine coating: A general strategy to enhance osteogenic differentiation and osseointegration for diverse implants. ACS Appl. Mater. Interfaces.

[18] Jiao X., Wang Z., Ma J., Wang T., Zhu D., Li H., Tang L., Li H., Wang C., Li Y. (2023). Polydopamine-coated 3D-printed β-tricalcium phosphate scaffolds to promote the adhesion and osteogenesis of BMSCs for bone-defect repair: mRNA transcriptomic sequencing analysis. J. Mater. Chem. B.

[19] Xing J., Wang Q., He T., Zhou Z., Chen D., Yi X., Wang Z., Wang R., Tan G., Yu P. (2018). Polydopamine-assisted immobilization of copper ions onto hemodialysis membranes for antimicrobial applications. ACS Appl. Bio Mater..

[20] Sui B., Xu Z., Xue Z., Xiang Y., Zhou T., Beltrán A.M., Zheng K., Liu X., Boccaccini A.R. (2023). Mussel-inspired polydopamine composite mesoporous bioactive glass nanoparticles: An exploration of potential metal-ion loading platform and in vitro bioactivity. ACS Appl. Mater. Interfaces.

[21] Kafkopoulos G., Martinho R.P., Padberg C.J., Duvigneau J., Wurm F.R., Vancso G.J. (2025). Metal Ions in Polydopamine Coatings Enhance Polymer-Metal Adhesion. ACS Appl. Polym. Mater..

[22] Sun H., Yang Y., Yu L., Liu K., Fei Y., Guo C., Zhou Y., Hu J., Shi L., Ji H. (2022). Inhibition of Inflammatory Response and Promotion of Osteogenic Activity of Zinc-Doped Micro-Arc Titanium Oxide Coatings. ACS Omega.

[23] Yu L., Guo Z., Yin Y., Sun H., Yu Y., Ni X., Ji H., Yang Y., Hu J. (2023). Preparation of Cerium-Containing Nanoporous Titanium Coatings for Osseointegration and Their Promotion of Osteogenic Differentiation and Anti-Inflammatory Properties. Mater. Today Chem..

[24] Zhang W., Wang G., Liu Y., Zhao X., Zou D., Zhu C., Jin Y., Huang Q., Sun J., Liu X. (2013). The Synergistic Effect of Hierarchical Micro/Nano-Topography and Bioactive Ions for Enhanced Osseointegration. Biomaterials.

[25] Li X., Xu H., Zhao B., Jiang S. (2018). Accelerated and Enhanced Osteointegration of MAO-Treated Implants: Histological and Histomorphometric Evaluation in a Rabbit Model. Int. J. Oral Sci..

[26] Chen J., Hao Y.L., Hou W.T., Wang H., Li C., Wang C., He Y. (2019). Effect of microarc oxidation-treated Ti6Al4V scaffold following low-intensity pulsed ultrasound stimulation on osteogenic cells in vitro. Ultrason. Sonochem..

[27] Wang S., Zhao X., Hsu Y., He Y., Wang F., Yang F., Yan F., Xia D., Liu Y. (2023). Surface modification of titanium implants with Mg-containing coatings to promote osseointegration. Acta Biomater..

[28] Yang F., Chang R., Webster T.J. (2019). Atomic layer deposition coating of TiO_2_ nano-thin films on magnesium-zinc alloys to enhance cytocompatibility for bioresorbable vascular stents. Int. J. Nanomed..

[29] Lee J.H., Mandakhbayar N., El-Fiqi A., Kim H.W. (2017). Intracellular co-delivery of Sr ion and phenamil drug through mesoporous bioglass nanocarriers synergizes BMP signaling and tissue mineralization. Acta Biomater..

[30] Ghanbari A., Bordbar-Khiabani A., Warchomicka F., Sommitsch C., Yarmand B., Zamanian A. (2023). PEO/Polymer Hybrid Coatings on Magnesium Alloy to Improve Biodegradation and Biocompatibility Properties. Surf. Interfaces.

[31] Mei Y., Zhu Y., Wei Y., Li S., Zhou X., Yao Y., Qiu J. (2025). Metal-Polydopamine Coordinated Coatings on Titanium Surface: Enhancing Corrosion Resistance and Biological Property. RSC Adv..

[32] Zhang J., Jiang S., Liu H., Wang Z., Cai X., Tan S. (2025). Fabrication of a ZnO/Polydopamine/ε-Polylysine Coating with Good Corrosion Resistance and a Joint Antibacterial Pathway on the Surface of Medical Stainless Steel. ACS Biomater. Sci. Eng..

[33] Ho M.H., Yao C.J., Liao M.H., Lin P.I., Liu S.H., Chen R.M. (2015). Chitosan Nanofiber Scaffold Improves Bone Healing via Stimulating Trabecular Bone Production Due to Upregulation of the Runx2/Osteocalcin/Alkaline Phosphatase Signaling Pathway. Int. J. Nanomed..

[34] Komori T. (2019). Regulation of Proliferation, Differentiation and Functions of Osteoblasts by Runx2. Int. J. Mol. Sci..

[35] Drevelle O., Daviau A., Lauzon M.A., Faucheux N. (2013). Effect of BMP-2 and/or BMP-9 on Preosteoblasts Attached to Polycaprolactone Functionalized by Adhesive Peptides Derived from Bone Sialoprotein. Biomaterials.

[36] Yuan C., Gou X., Deng J., Li T., Li J., Hu L., Yang H., He Y., Wang Y., Wang Z. (2018). FAK and BMP-9 Synergistically Trigger Osteogenic Differentiation and Bone Formation of Adipose Derived Stem Cells Through Enhancing Wnt-β-Catenin Signaling. Biomed. Pharmacother..

[37] Wang S., Xia D., Dou W., Chen A., Xu S. (2024). Bioactive Porous Composite Implant Guides Mesenchymal Stem Cell Differentiation and Migration to Accelerate Bone Reconstruction. Int. J. Nanomed..

[38] Zhou J., Zhao L., Li B., Han Y. (2018). Nanorod Diameter Modulated Osteogenic Activity of Hierarchical Micropore/Nanorod-Patterned Coatings via a Wnt/β-Catenin Pathway. Nanomedicine.

[39] Li K., Hu D., Xie Y., Huang L., Zheng X. (2018). Sr-Doped Nanowire Modification of Ca-Si-Based Coatings for Improved Osteogenic Activities and Reduced Inflammatory Reactions. Nanotechnology.

[40] Pei D., Zeng Z., Geng Z., Zhang Y., Li J., Li Y., Li G., Cai H., Wang Q., Li B. (2024). Modulation of Macrophage Polarization by Secondary Cross-Linked Hyaluronan-Dopamine Hydrogels. Int. J. Biol. Macromol..

[41] Wu T., Liu W., Huang S., Chen J., He F., Wang H., Zheng X., Li Z., Zhang H., Zha Z. (2021). Bioactive strontium ions/ginsenoside Rg1–incorporated biodegradable silk fibroin-gelatin scaffold promoted challenging osteoporotic bone regeneration. Mater. Today Bio.

[42] Chen T., Wu X., Zhang P., Chen H., Zhang X., Zhang W., Cai Z., Zhang S., Zhang Y., Wang W. (2024). Strontium-Doped Hydroxyapatite Coating Improves Osteo/Angiogenesis for Ameliorative Graft-Bone Integration via the Macrophage-Derived Cytokines-Mediated Integrin Signal Pathway. ACS Appl. Mater. Interfaces.

[43] Sun T., Li Z., Zhong X., Cai Z., Ning Z., Hou T., Xiong L., Feng Y., Leung F., Lu W.W. (2019). Strontium Inhibits Osteoclastogenesis by Enhancing LRP6 and β-Catenin-Mediated OPG Targeted by MiR-181d-5p. J. Cell Commun. Signal..

[44] Zhu S., Hu X., Tao Y., Ping Z., Wang L., Shi J., Wu X., Zhang W., Yang H., Nie Z. (2016). Strontium Inhibits Titanium Particle-Induced Osteoclast Activation and Chronic Inflammation via Suppression of NF-κB Pathway. Sci. Rep..

[45] Roux C., Fechtenbaum J., Kolta S., Fardellone P., Jackson R.D., Adami S., Mitlak B.H. (2008). Strontium Ranelate Reduces the Risk of Vertebral Fracture in Young Postmenopausal Women with Severe Osteoporosis. Ann. Rheum. Dis..

[46] Deeks E.D., Dhillon S. (2010). Strontium Ranelate: A Review of Its Use in the Treatment of Postmenopausal Osteoporosis. Drugs.

[47] Li J., Zhang Y., Chen H., Wang Y., Zhang X., Liu Y., Wang S., Xia D., Dou W., Chen A. (2023). Molecular Mechanisms of Strontium in Regulating Bone Metabolism and Its Therapeutic Potential in Osteoporosis. Bioact. Mater..

