# Supplementary material for: Osteogenic and Anti-Inflammatory Effects of Strontium-Loaded Polydopamine on Micro-Arc Oxidized Titanium Surfaces
Source: J Funct Biomater. 2026 Apr 7;17(4):181. doi: 10.3390/jfb17040181 (PMC13118214; doi:10.3390/jfb17040181)
Supplement: Supplementary file 1 [file jfb-17-00181-s001.zip › jfb-4191875-supplementary.pdf]

Supplementary Data for

Osteogenic and Anti-Inflammatory Effects of Strontium-Loaded Polydopamine on Micro-Arc Oxidized Titanium Surfaces

Yiming Yang et al.

Correspondence: fangbing@sjtu.edu.cn(B.F.); lqlqliaoqian@hotmail.com(Q.L.);

This PDF file includes:

Table S1. Primers for the Expression of Osteogenesis-Related Genes in rBMSCs.

Gene	Forward Primer Sequence	Reverse Primer Sequence
Runx2	ACAACCACAGAACCACAAG	TCTCGGTGGCTGGTAGTGA
Osterix	CGGCAAGGTGTACGGCAAGG	GAGCAGAGCAGACAGGTGAACTTC
ALP	GGGACTGGTACTCGGACAAT	GGCCTTCTCATCCAGTTCAT
OCN	GGTGCAGACCTAGCAGACACCA	AGGTAGCGCCGGAGTCTATTCA
Col1	CATGTTCAGCTTTGTGGACCT	GCAGCTGACTTCAGGGATGT
OPN	GACGATGATGACGACGACGATGAC	GTGTGCTGGCAGTGAAGGACTC
β-actin	CTTTCTACAATGAGCTGCGTG	ATGGCTGGGGTGTTGAAGG

Table S2. Primers for the Expression of f Inflammation-Related Genes in RAW264.7.

Gene	Forward Primer Sequence	Reverse Primer Sequence
iNOS	GGTGAAGGGACTGAGCTGTT	ACGTTCTCCGTTCTCTTGCAG
CD86	AAAGAGGAGCAAGCAGACGC	CTCCACGGAAACAGCATCTGAG
CCR7	TCTCCTTGTCATTTTCCAGGTGT	CTTGCTGATGAGAAGCACGC
TNF-α	CCCACGTCGTAGCAAACCAC	TAGCAAATCGGCTGACGGTG
CD163	ACTTCTCAGTGCCTCTGCTG	TCTCCAAATTAGATCCATCTGAGC
CD206	GATGACCTGTGCTCGAGAGG	TCTCGCTTCCCTCAAAGTGC
Arg-1	TTCTCAAAAGGACAGCCTCG	GGCCAGAGATGCTTCCAACT
BMP-2	AACGAGAAAAGCGTCAAGCC	AGGTGCCACGATCCAGTCAT
GAPHD	ACTCAGGAGAGTGTTTCCTCG	TTTGCCGTGAGTGGAGTCAT

Table S3. Primers for the Expression of Osteoclast-Related Genes in mice.

Gene	Forward Primer Sequence	Reverse Primer Sequence
CTSK	GGGAGAAAAACCTGAAGC	ATTCTGGGGACTCAGAGC
DC-STAMP	AAAACCCTTGGGCTGTTCTT	AATCATGGACGACTCCTTGG
TRAP	CTGGAGTGCACGATGCCAGCGACA	TCCGTGCTCGGCGATGGACCAGA
c-Fos	CCAGTCAAGAGCATCAGCAA	AAGTAGTGCAGCCCGGAGTA
GAPHD	GGTGAAGGTCGGTGTGAACG	CTCGCTCCTGGAAGATGGTG

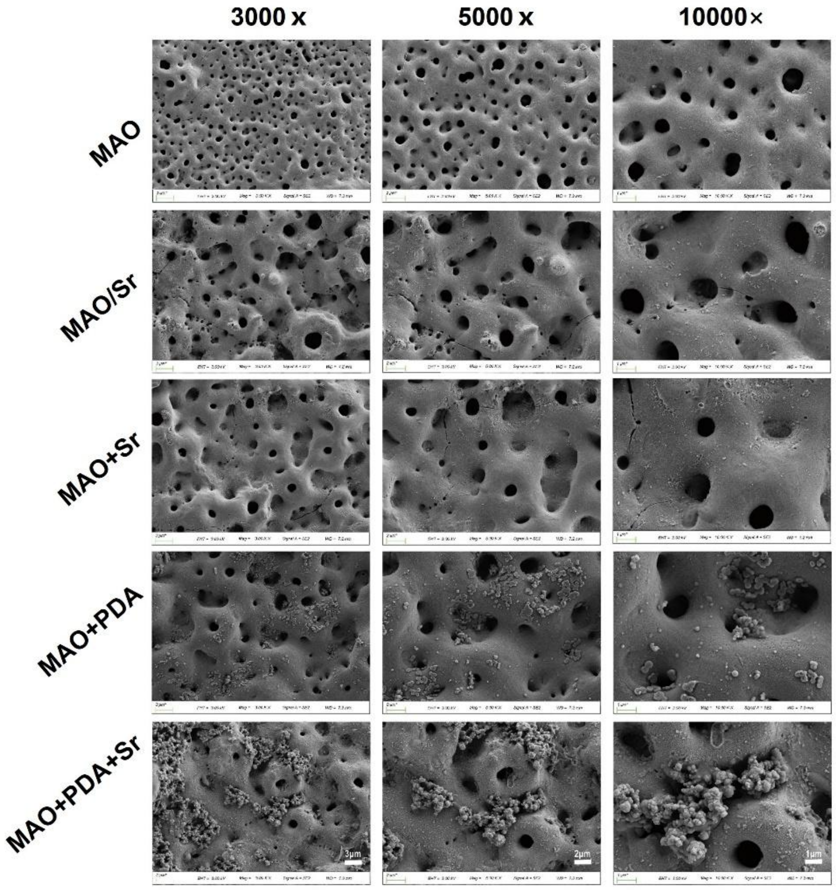

Figure S1. Representative SEM images showing the surface morphologies of different coating materials under various background conditions at magnifications of 3000×, 5000× and 10,000×, respectively.

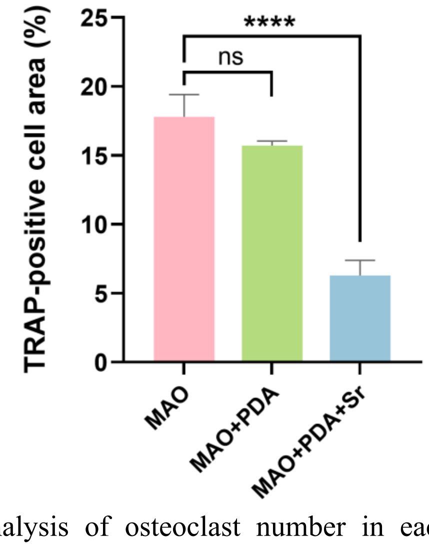

Figure S2. Quantitative analysis of osteoclast number in each group stained by TRAP. Statistical significance was determined using one-way ANOVA with Dunnett's post hoc test. ****$p < 0.0001$; ns, not significant.
